# Proteomic analysis reveals a potential role for extracellular vesicles within the erythroblastic island niche

**DOI:** 10.3389/fmolb.2024.1370933

**Published:** 2024-04-16

**Authors:** Telma Ventura, Antonella Fidanza, Marieangela C. Wilson, Daniel C. J. Ferguson, Phillip A. Lewis, Alisha May, Helen Taylor, Michael P. Rimmer, Christopher D. Gregory, Jan Frayne, Lesley M. Forrester

**Affiliations:** ^1^ Centre for Regenerative Medicine, Institute for Regeneration and Repair, University of Edinburgh, Edinburgh, United Kingdom; ^2^ Edinburgh Medical School, Biomedical Sciences, University of Edinburgh, Edinburgh, United Kingdom; ^3^ Proteomics Facility, Faculty of Life Sciences, University of Bristol, Bristol, United Kingdom; ^4^ School of Biochemistry, University of Bristol, Bristol, United Kingdom; ^5^ Centre for Inflammation Research, Institute for Regeneration and Repair, University of Edinburgh, Edinburgh, United Kingdom

**Keywords:** erythroblastic island macrophage, extracellular vesicles, induced pluripotent stem cells, proteomics, KLF1

## Abstract

**Introduction:** Erythroblastic island (EBI) macrophages play an essential role in the production and maturation of the vast numbers of red blood cells (RBCs) that are produced throughout life. Their location within the bone marrow makes it difficult to study the cellular and molecular interactions associated with their action so we have used an *in vitro* model of the EBI niche using macrophages derived from human induced pluripotent stem cells (hiPSCs). We previously demonstrated that the activation of the transcription factor KLF1 enhanced the activity of hiPSC-derived EBI macrophages.

**Methods:** To elucidate the mechanisms associated with EBI-like activity we carried out a quantitative proteomic analysis and assessed the role of extracellular vesicles using Nanosight Tracking analyses and media filtration.

**Results and Discussion:** Gene ontology analysis showed that many of the proteins upregulated by KLF1 were protein-binding factors, some of which were associated with the cell membrane or extracellular vesicles We demonstrated that filtration of macrophage-conditioned media resulted in a reduction in the supportive effects on erythroid cell viability and maturation implying a role for extracellular vesicles but this was not KLF1 dependent. Pathway analyses of the proteomic data revealed that proteins upregulated by KLF1 were associated with the citric acid cycle, pyruvate metabolism and ATP synthesis indicating that KLF1-activated macrophages had a metabolic profile comparable to a pro-reparative phenotype. This study has generated a proteomic dataset that could provide new insights into the role of macrophages within the EBI niche and has indicated a potential role for extracellular vesicles in the differentiation and maturation of RBCs *in vitro*. Further research will aid in the production of RBCs *in vitro* for use in disease modelling and cell therapy.

## Introduction

The production and maturation of red blood cells (RBCs) takes place within the erythroblastic island (EBI) niche where central macrophages provide support for proliferating and maturing erythroid cells (Lopez-Yrigoyen et al., 2019; Li et al., 2020; May and Forrester, 2020; May et al., 2023). The inaccessible location of this important niche within the bone marrow makes it difficult to study so the cellular and molecular mechanisms associated with its function remain poorly understood. Several model systems have been used to study the EBI niche including relatively simple co-culture models *in vitro* and sophisticated genetic depletion strategies *in vivo* (Chow et al., 2011; Heideveld et al., 2015; May and Forrester, 2020; Romano et al., 2022). The *ex-vivo* proliferation and differentiation of human erythroid progenitor cells were significantly enhanced when co-cultured with macrophages derived from various sources including from peripheral blood mononuclear cells and from human induced pluripotent stem cells (hiPSCs) (Heideveld et al., 2015; Lopez-Yrigoyen et al., 2019; May et al., 2023). This provided a strategy to study the molecular processes that are associated with the EBI niche. The ease with which hiPSCs can be genetically manipulated makes this source particularly powerful. We previously demonstrated that hiPSC-derived macrophages could be genetically programmed to an EBI-like phenotype using the Erythroid Krüppel-Like transcription factor, EKLF/KLF1. When KLF1 was activated in macrophages their ability to support the proliferation and maturation of umbilical cord blood (UCB)-derived erythroid progenitor cells was significantly enhanced, and we identified downstream targets of KLF1 that were candidates to be involved in macrophage-RBC interactions (Lopez-Yrigoyen et al., 2019). Some of the enhanced activity was retained when cell-cell interactions were inhibited in a trans-well assay and this led to the identification and validation of genes encoding secreted factors, ANGPTL7, IL33 and SERPINB2. That study also identified genes encoding membrane associated proteins that would be predicted to be involved in inter-cellular interactions but the translation of these transcripts into functional proteins was not confirmed nor characterised. Here we have used a proteomic approach to identify proteins that are expressed within EBI macrophages and proteins that are present at higher levels upon activation of KLF1. Some of these proteins were associated with the cell membrane or extracellular vesicles, thus, we explored whether the production and release of extracellular vesicles (EVs) played a role in the communication between EBI macrophage and maturing RBCs. EVs include exosomes which originate from the endosome system and microvesicles that are shed from the plasma membrane and represent an alternative mechanism for intercellular communication, allowing cells to exchange proteins, lipids and genetic material (Doyle and Wang, 2019; Wang et al., 2020). EVs derived from macrophages have been shown to have diverse roles as mediators of the inflammatory response as well as tissue pathology and repair (Wang et al., 2020) but to our knowledge no studies on EVs within the EBI niche have been reported. Here, we demonstrate that the exclusion of EVs from macrophage-conditioned media resulted in a reduction in its supportive effect corroborating the hypothesis that EVs play an important role in intracellular communication within the EBI niche.

## Methods

### Macrophage culture

SFCi55 and iKLF1.2 hiPSCs were maintained and the differentiation of macrophages was performed as previously described (Yang et al., 2017; Lopez-Yrigoyen et al., 2019; Lopez-Yrigoyen et al., 2020). Briefly hiPSCs were differentiated into embryoid bodies in the presence of 50 ng/mL VEGF, 50 ng/mL BMP4 and 20 ng/mL SCF for 6 days then plated in 25 ng/mL IL3 and 100 ng/mL M-CSF for at least 16 days with media being changed every 3 days. Suspension cells were collected at different time points then macrophages were matured for up to 10 days in the presence of 100 ng/mL m-CSF (macrophage maturation media). KLF1 was activated in iKLF1.2 macrophages with the addition of 200 nM of tamoxifen at days 7 and 9 of maturation.

### Umbilical cord blood-derived CD34^+^ cell culture

Umbilical cord blood (UCB)-derived CD34^+^ cells (Stemcell Technologies, 70008.3) were thawed, expanded and differentiated into erythroblasts as described (Douay and Giarratana, 2009; Griffiths et al., 2012). Briefly CD34^+^ cells were first expanded for 6 days in ISHI media [IMDM (Gibco), 3U/mL Heparin (Merck), 10 μg/mL Insulin (Sigma), 200 μg/mL of holo-transferrin (Merck) and 5% Human AB serum (Sigma)) in the presence of cytokine cocktail A (60 ng/mL of SCF (Life Technologies), 5 ng/mL of IL3 (Peprotech) and 3U/mL of EPO (R&D Bio-Techne)]. These expanded cells were cryopreserved in batches of 10^6^ cells/mL in a 1:1 mix of ISHI media and freezing media (60% KnockOut Serum Replacement (Gibco), 20% DMSO (Invitrogen) and 20% ISHI media). These “day 6” cells were thawed and used as a starting point for the erythroid differentiation in macrophage co-culture experiments.

### Flow cytometry

Cells for flow cytometry analysis were resuspended in PBS with 1% BSA (A2153, Sigma-Aldrich) and 5 mM EDTA (15575020, Invitrogen). 10^5^ cells per sample were stained with appropriate antibodies for 15 min at room temperature and with Hoechst dye as previously described (Lopez-Yrigoyen et al., 2019). Data was collected and analysed using the LSR Fortessa (BD Biosciences) with BD FACSDiva and FlowJo 10.8.1 software. Briefly, single and live cells were gated and FMO controls were used to distinguish specifically stained cell populations positive. All antibodies used are listed in Supplementary Table S1.

### Immunofluorescence

Immunofluorescence of macrophages was performed by collecting 6 × 10^4^ suspension cells and seeding them on a μClear^®^ cell culture 96-well plate for 10 days. iKLF1.2 macrophages were treated with tamoxifen twice at days 7 and 9 of maturation. At day 10 mature macrophages were washed twice with DPBS (Gibco) and fixed with 4% PFA for 10 min at room temperature. Cells were then permeabilised with DPBS + Triton-X100, 0.1% (DPBST) twice for 10 min, and blocked for 2 h with blocking solution (DPBST +1% BSA +3% Donkey Serum). Cells were then incubated with goat anti-EKLF/KLF1 antibody (Thermo, cat. PA5-18031) at a dilution of 1:200 in blocking solution overnight at 4°C. Cells were washed with PBST three times for 15 min and incubated with the secondary antibody, donkey anti-goat AF647, (1:1000) and DAPI (1:1000) in blocking solution for 2 h at room temperature in the dark. Cells were washed three times with DPBST for 15 min and DPBS was added before imaging. Images were taken with Zeiss Observer Microscope and Axion Vision software.

### Gene expression analyses

RNA extraction was performed using the RNAeasy Mini Kit (74106, QIAGEN) following the manufacturer’s instructions and cDNA was generated as previously described (May et al., 2023). Quantitative real time PCR reactions were performed on the Roche LightCycler^®^ 480 Instrument. 2 ng of cDNA was amplified with LightCycler^®^ 480 SYBR Green I Master (4887352001, Roche) according to the manufacturer’s instructions. All reactions were performed with 3 biological and 3 technical replicates. Ct values were normalised to the reference gene *GAPDH* or the mean of the reference genes *GAPDH* and *β-Actin* as indicated. Data was analysed using the 2^−ΔΔCT^ method and graphs were generated and statistical analysis was performed using GraphPad Prism 8 software. Primers used are listed in Supplementary Table S2.

### Quantitative proteomics

For protein extraction, hiPSCs-derived macrophages (3 × 10^6^ cells/mL per sample) were washed twice with ice cold DPBS and detached with 200ul of Radioimmunoprecipitation assay (RIPA) buffer (25 mM Tris-HCl pH7.6, 150 nM NaCl, 1% NP-40, 1% Sodium deoxycholate, 0.1% SDS) (Thermo Scientific) and Protease inhibitor cocktail (Sigma), incubated on ice for 30 min and vortexed every 10 min. Samples were sonicated (Diagenode Bioruptor^®^ Pico) three times for 30 s in 30 s intervals, centrifuged for 15 min at 14000 g at 4°C and protein concentration of the supernatant was quantified using a Pierce Rapid Gold BCA Protein Assay Kit (Thermo Scientific) protein quantification assay. Lysates were snap frozen and stored at −80°C until analysis.

Quantitative proteomic analysis of hiPSC-derived macrophages was performed using Tandem Mass Tagging (TMT) as previously described (Daniels et al., 2021; Ferrer-Vicens et al., 2023). Briefly, 100 mg lysates were labelled with TMT 11-plex reagents (Thermo Scientific). An equal amount of all samples was pooled and used as reference during analysis. All spectra were acquired using Orbitrap Fusion Tribrid mass spectrometer on Xcallibur 2.0 software. Raw data files were processed and quantified using Proteome Discoverer v2.1 software (Thermo Scientific). Proteins were identified by searching against Uniprot Human database and Common Contaminants database, using Sequest^®^ algorithm. For the ‘Total Proteome’ analysis, each sample analysed was normalised on the ‘Total Peptide Amount’ and filtered using a 5% false discovery rate (FDR). The mass spectrometry proteomics data have been deposited to the ProteomeXchange Consortium via the PRIDE [1] partner repository with the dataset identifier PXD050357.

### Biological function and pathway analysis of proteins

Network of biological and pathway analysis of differently expresses proteins were assessed using Web-based gene set analysis toolkit (WebGestalt) (https://www.webgestalt.org/, accessed September 2023) (Liao et al., 2019) and Reactome (https://reactome.org/, accessed September 2023). Using WebGestalt the differently expressed proteins were annotated against the identified proteins (ID mapped) in their database, from which gene ontology (GO) and over-representation analysis (ORA) were performed.

### Preparation of complete and EV-depleted media

To prepare conditioned media, ISHI media was double filtered with PVDF membrane low protein binding filters with 0.22 μm (E4780-1221, Starlab) and 0.1 μm (SLVV033RS, Millipore) pore size, and was added to macrophages at day 7 of maturation (with 100 ng/ml m-CSF). Where appropriate filtered tamoxifen was added to macrophage conditioned media at a concentration of 200 nM. On day 10 of macrophage maturation, the media in contact with macrophages was collected, passed through a 5 μm strainer (43-50005-13, Pluriselect) then centrifuged at 300 *g* for 5 min to exclude cell debris. To subsequently deplete EVs, the conditioned media collected from macrophages was again double filtered with 0.22 μm (Starlab) and 0.1 μm low protein attachment syringe filters (Millipore).

### Erythroid differentiation in macrophage conditioned media

For erythroid differentiation, day 6 UCB-derived erythroblasts were thawed and cultured in ISHI media in the presence of cytokine cocktail A until day 7 of differentiation (see above). At day 8 cells were centrifuged and cultured until day 10 in filtered and unfiltered macrophage condition media in the presence of cytokine cocktail B (60 ng/mL of SCF, 3U/mL of EPO, 1uM of Hydrocortisone (Stemcell Technologies) and 300 μg/mL of holo-transferrin (Merck)). From day 11 to day 21 of differentiation cells were cultured in either filtered and unfiltered macrophage conditioned media in the presence of cytokine cocktail C (3U/mL of EPO and 300 μg/mL of holo-transferrin).

### Nanosight tracking analyses

Extracellular vesicles produced by hiPSCs-derived macrophages were analysed using Nanosight LM10 with an sCMOS camera (Malvern, Panalytical, United Kingdom). Collected media from macrophages was vigorously vortexed and injected in the Nanosight chamber 300 μL at a time using a 1 mL syringe and data was recorded for 3 × 30 s videos at 22°C, under media viscosity of 0.953 centipoise (cP). The number, size and concentration of particles in each sample was assessed using NTA software 2.3 (build 0011 RC1).

## Results

**FIGURE 1:**
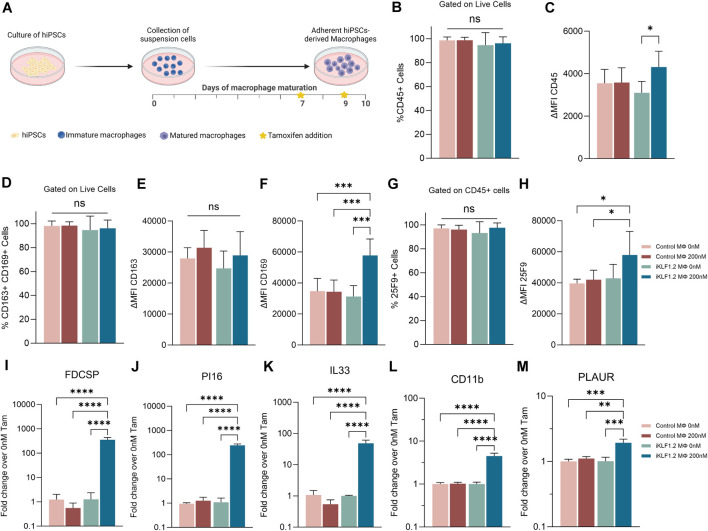
KLF1 activation enhances the maturation of iPSC-derived macrophages. **(A)** Schematic diagram of protocol used to differentiate and mature macrophages from iPSCs showing the timing of tamoxifen addition in relation to days of differentiation. B-H. Flow cytometry analyses of macrophages derived from SFCi55 iPSCs (Control MΦ) or iKLF1.2 iPSCs (iKLF1 MΦ) cultured without (0 nM) or with (200 nM) tamoxifen showing the percentage of cells that express CD45 **(B)**, CD163 and CD169 **(D)** or 25F9 **(G)** and the level of expression (measured by the mean fluorescent intensity (MFI)) of CD45 **(C)**, CD163 **(E)**, CD169 **(F)** and 25F9 **(H)**. **(I–M)** Quantitative RT-PCR analyses of RNA derived from SFCi55 iPSCs (Control MΦ) or iKLF1.2 iPSCs (iKLF1 MΦ) macrophages cultured without (0 nM) or with (200 nM) tamoxifen using primers designed to amplify *FDCSP, PI16, IL33, CD11b, PLAUR* and *CD169*. N = 3 independent experiments, statistical test: 2-way ANOVA, with Tukey multiple comparison. *p < 0.05, **p < 0.01, ***p < 0.005, ****p < 0.001. The schematic diagram in A was created using Biorender.

Macrophages were differentiated from control hiPSCs and hiPSCs that had been genetically manipulated to insert the CAG*-KLF1-ER* ^*T2*^ cassette within the *AAVS1* locus (Figure 1A) (Yang et al., 2017; Lopez-Yrigoyen et al., 2020). Two days prior to the harvesting of macrophages, 200 nM tamoxifen was added to the culture media to activate translocation of the KLF1-ER^T2^ protein from the cytoplasm to the nucleus (Supplementary Figure S1A). Macrophages derived from control and iKLF1.2 hiPSCs had comparable morphology (Supplementary Figure S1B). Flow cytometry analyses demonstrated that the viability of macrophages derived from iKLF1.2 and control hiPSCs in the presence and absence of tamoxifen were comparable (data not shown). The proportion of live cells expressing various cell surface markers was assessed by flow cytometry with the level of expression of these markers being based on the mean fluorescent intensity (MFI) (Figures 1B–H). Almost all macrophages derived from both control and iKLF1.2 hiPSC lines expressed the pan haematopoietic marker, CD45 (Figure 1B) and we noted a slightly higher level of expression in macrophages derived from iKLF1.2 hiPSCs when KLF1 was activated with 200 nM tamoxifen (Figure 1C). Almost all macrophages also expressed macrophage markers CD163 and CD169 (Figure 1D) and this was unaffected by the addition of tamoxifen. Tamoxifen treatment also had no effect on the level of expression of CD163 (Figure 1E), but activation of KLF1 in iKLF1.2-derived macrophages resulted in a significantly higher level of expression of CD169 (Figure 1F). Expression of the macrophage marker 25F9 reflects the stage of maturation and whereas almost all macrophages expressed 25F9, we noted an increase in the level of expression upon tamoxifen addition in iKLF1.2 macrophages (Figures 1G,H). These data are consistent with our previous report that the activation of KLF1 enhances overall macrophage maturation (Lopez-Yrigoyen et al., 2019).

To further confirm the effects of KLF1 activation in the hiPSC-derived macrophages used in this study, we analysed the expression of genes that we previously identified as KLF1 targets by real time qPCR (Lopez-Yrigoyen et al., 2019). Following addition of tamoxifen, the level of expression of *FDCSP, PI16, IL33, CD11B* and *PLAUR* were all upregulated upon addition of tamoxifen in macrophages derived from iKLF1.2 but not control hiPSCs (Figures 1I–M) consistent with our previous findings (Lopez-Yrigoyen et al., 2019).

### Proteomic analyses

**FIGURE 2:**
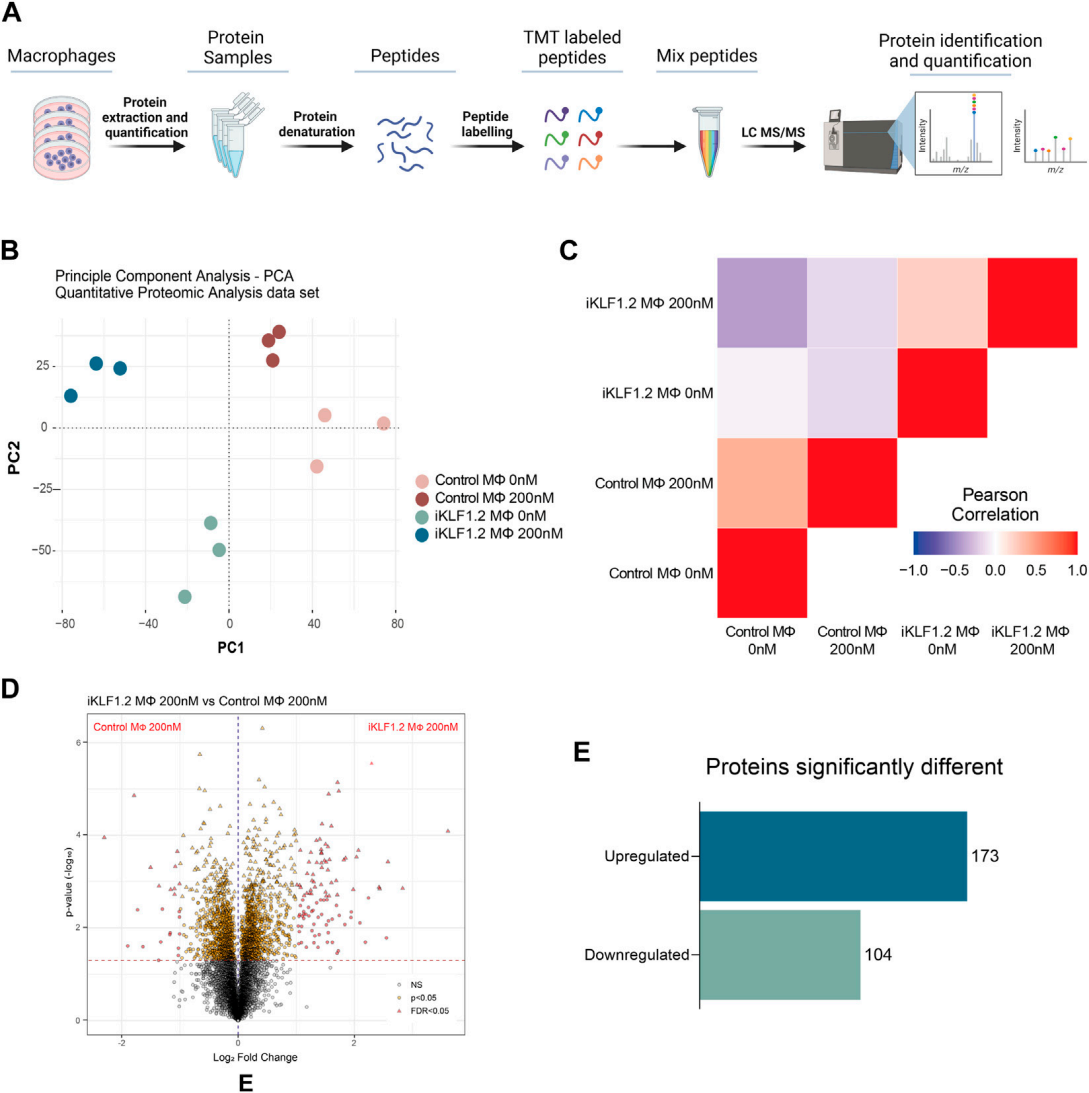
Proteomic analyses of KLF1-activated iPSC-derived macrophages. **(A)** Schematic diagram of process associated with Proteomic analyses of iPSC-derived macrophages including extraction, quantification, denaturation, labelling and identification by tandem mass spectrometry (TMT). **(B)** Principal Component analyses of proteins identified in macrophages derived from SFCi55 iPSCs (Control MΦ) or iKLF1.2 iPSCs (iKLF1 MΦ) cultured without (0 nM) or with tamoxifen (200 nM). **(C)** Pearson correlation analyses of proteins in samples described in **(B)**. **(D)** Volcano plots comparing proteins present in KLF1 200 nM vs. Parental 200 nM samples. **(E)** Number of upregulated and downregulated proteins in KLF1 activated macrophages, 173 and 104, respectively, after data-set triage. The schematic diagram in A was created using Biorender.

To assess whether our previous reported KLF1-related transcriptomic remodelling was translated into changes in the proteome, we carried out quantitative proteomic analyses of control and iKLF1.2 hiPSC-derived macrophages in the presence and absence of 200 nM tamoxifen (Figure 2A). Protein profiling was performed using Tandem Mass Tagging (TMT) Quantitative Proteomic analysis as previously described (Daniels et al., 2021; Ferrer-Vicens et al., 2023).

Principle Component Analysis (PCA) of the 7,868 independent proteins that were quantified showed that the different samples and their respective replicates fell into distinct clusters that did not overlap (Figure 2B). Pearson Correlation analyses demonstrated that samples derived from iKLF1.2 hiPSCs in the presence and absence of tamoxifen were more closely related to each other than the equivalent samples derived from control hiPSCs indicating some cell line-specific differences (Figure 2C).

**FIGURE 3:**
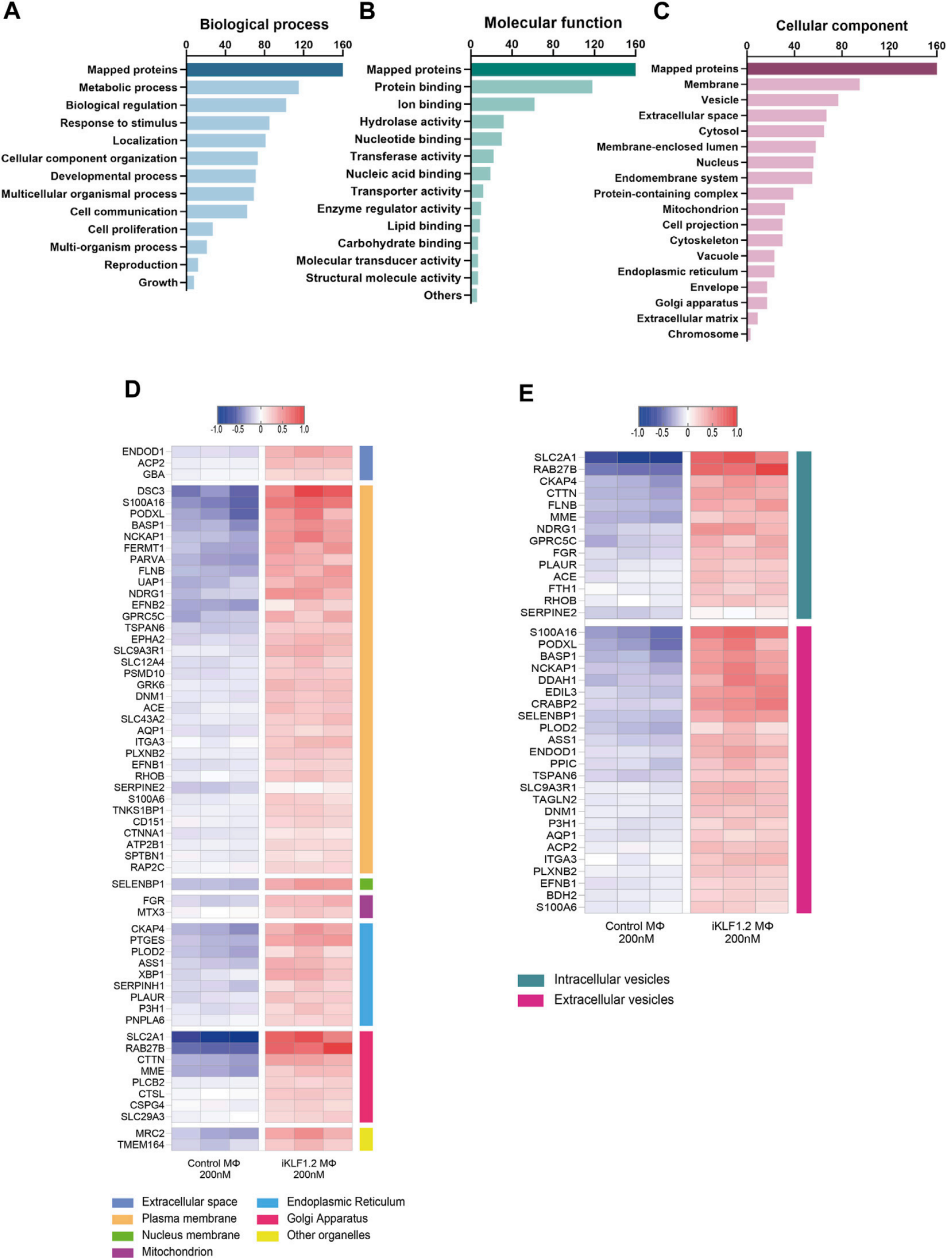
Gene Ontogeny analyses of proteins upregulated by KLF1 in iPSC-derived macrophages. Gene ontogeny (GO) analysis of the proteins that are upregulated by KLF1 showing proteins associated with different biological processes **(A)** molecular functions **(B)** and cellular components **(C)**. Heatmaps of proteins associated with membrane complexes and extracellular vesicles **(D,E)**.

**FIGURE 4:**
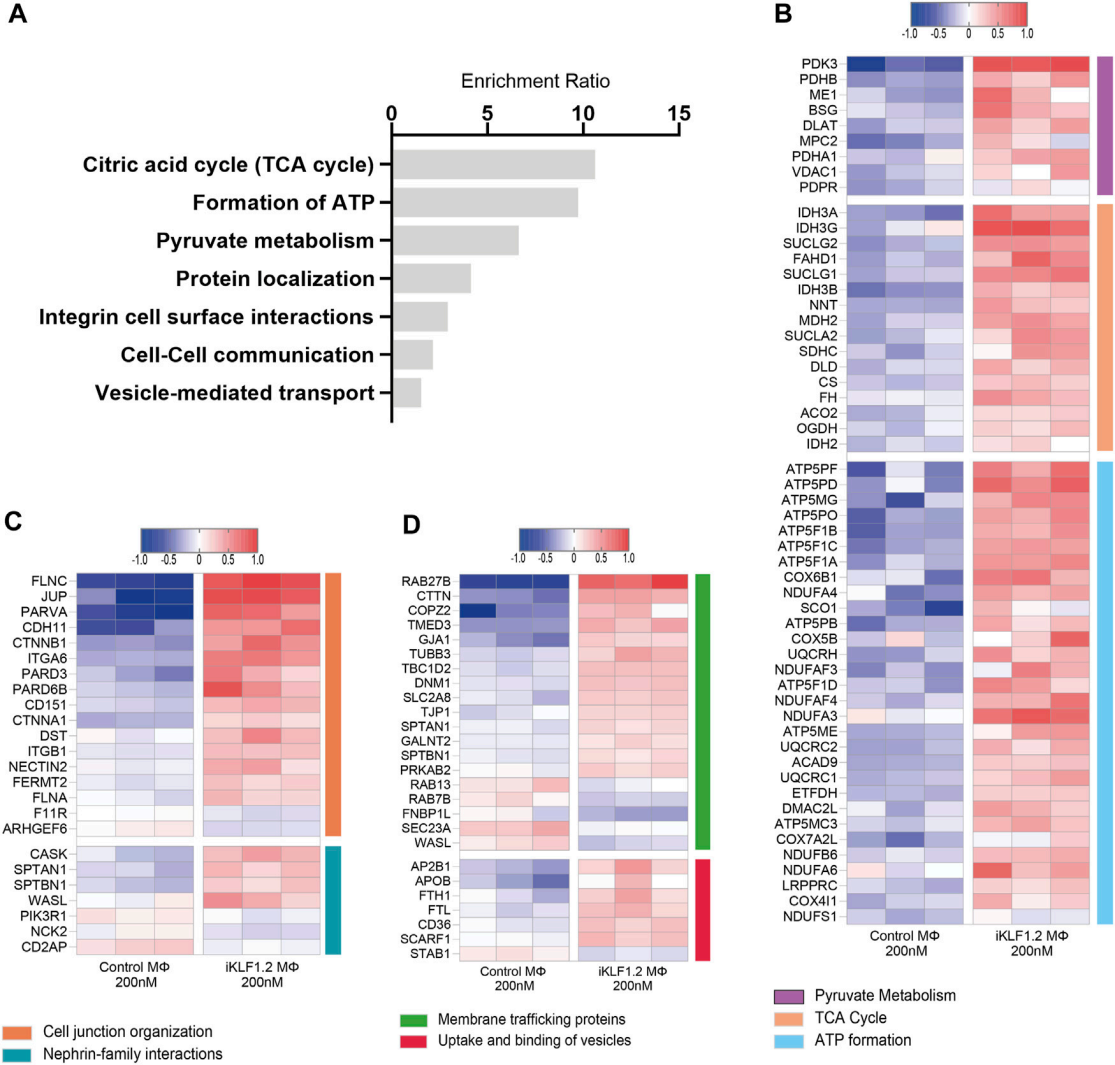
Biological pathways associated with KLF1-regulated proteins. **(A)** Enrichment Ratio (ORA) of pathway analyses revealing that the most significant pathways of upregulated proteins are associated with metabolic processes. **(B)** Heatmap of the top differentially expressed proteins that are associated with metabolic processes. **(C,D)** Heatmaps of the top differentially expressed proteins that are associates with cell junctions, trafficking and the uptake and binding of vesicles.

Volcano plots of data generated from control and iKLF1.2 hiPSCs-derived macrophages in the absence of tamoxifen also revealed underlying intrinsic proteomic differences between cell lines (Supplementary Figures S2A–S2C). Volcano plots also revealed proteins that were regulated by tamoxifen alone which were proteins that were differentially expressed in macrophages derived from control hiPSCs in the presence and absence of tamoxifen (Supplementary Figure S2B). As expected, more differentially expressed proteins were identified when comparing macrophages derived from iKLF1.2 hiPSCs in the presence and absence of tamoxifen (Supplementary Figure S2C). We anticipated that this cohort of proteins would include proteins that were differentially expressed due to the activation of KLF1 as well as those that were induced by tamoxifen alone. We therefore considered that the most meaningful and biological relevant proteins would be those that were differentially expressed when comparing tamoxifen-treated macrophages derived from control and iKF1.2 hiPSCs (Figure 2D). This comparison identified 2051 differentially expressed proteins and following the application of a false discovery rate of 0.05, 321 differentially expressed proteins were identified. 44 of these proteins were eliminated as they were also identified as being activated by tamoxifen in macrophages derived from control hiPSCs. A final list of 277 proteins that were differentially expressed upon the activation of KLF1 in hiPSC-derived macrophages were then used in subsequent analyses. Of these, 173 proteins were significantly upregulated and 104 were downregulated (Figure 2E). Bioinformatic analyses were carried out proteins that were both KLF1-upregulated (Figures 3, 4) and KLF1 downregulated proteins (Supplementary Figures S4, S5). However, in this study we focussed our attention on the proteins that were upregulated by KLF1 as we considered these to be more likely to be associated with erythroblastic island macrophages.

Gene ontogeny analyses of the upregulated proteins using the web-based tool WebGestalt showed that 160 out of the 173 upregulated proteins were unambiguously identified (mapped), with 13 proteins unmapped due to their lack of function according to the protein database. For these mapped proteins those associated with metabolism was the top category within biological processes; protein binding was the top category within molecular functions and cell membrane and vesicles was top within the cellular components category (Figures 3A–C). As our key interest was in the communication of the macrophages within the EI niche with differentiating erythroid cells, we carried out further analyses on the upregulated proteins that were associated with membranes and vesicles. We annotated the sub-cellular localisation of these proteins and found that many of these proteins are found in the plasma membrane, suggesting a possible involvement in cell-to-cell communication (Figure 3D). Many of the KLF1 upregulated proteins associated with vesicles are associated with extracellular vesicles, including RAB27B which could be responsible for the transport of specific cargo relevant to cell communication (Figure 3E).

Over-representation analyses (ORA) analysis was also used to identify the most enriched biological pathways associated with differentially expressed genes (Figure 4A). The expression of all proteins associated with these enriched pathways was then assessed and we noted the upregulation of proteins associated with the citric acid cycle, formation of ATP and pyruvate metabolism implying that the KLF1 activation alters the metabolic profile of macrophages (Figure 4B). In keeping with the GO analyses, ORA analyses also revealed a significant enrichment of proteins associated with cell-cell communication and vesicle-mediated transport (Figure 4A). Further analyses of proteins in these categories in our dataset revealed that KLF1 upregulated proteins included those associated with cell junctions, membrane trafficking and the uptake and binding of vesicles (Figures 4C,D). This set of proteins was of particular interest because it is possible that they could be involved in the communication between macrophages and erythroid cells within the EBI niche. Although we had previously predicted that both secreted factors and direct cell contact were involved in communication, we had not considered nor explored the role of extracellular vesicles.

### Macrophage-derived extracellular vesicles play a role in the EBI niche

We previously reported that the communication between macrophages and differentiating erythroid cells involved direct cell-cell interaction as well as factors present in the media that could cross the membrane of a trans-well culture system. In that study we demonstrated that the addition of a few selected recombinant proteins (ANGPTL7, IL33 and SERPINB2) had an enhancing effect on erythroid maturation (Lopez-Yrigoyen et al., 2019). However, we had not assessed the effect of the complete conditioned media from control nor KLF1-activated macrophages. The content of that conditioned media would be predicted to include secreted factors as well as extracellular vesicles (EVs). EVs derived from macrophages have been shown to be associated with the inflammatory response and tissue repair (Wang et al., 2020) but to our knowledge there have been no studies on EVs derived from macrophages within the EBI niche. Given that some of the proteins identified in our proteomic screen were associated with extracellular vesicles (EVs) we set out to tested whether this form of intracellular communication may play a role in this system.

**FIGURE 5:**
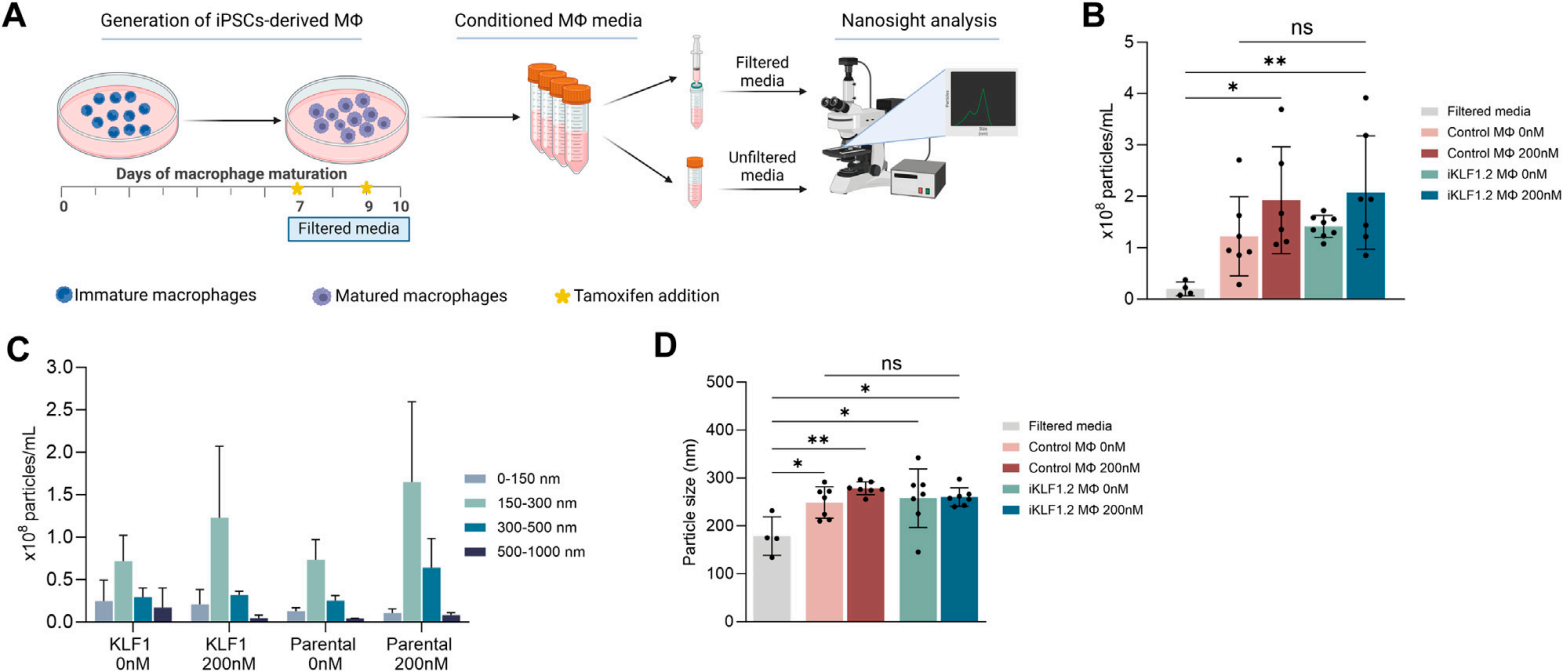
Characterisation of particles in macrophage conditioned media by Nanosight technology. **(A)** Schematic diagram of unfiltered and filtered conditioned media preparation and particle analyses using Nanocyte technology. Importantly all cells were switched to pre-filtered media immediate prior to the experimental setup. **(B)** Concentration of particles present in unfiltered media derived from macrophages generated from SFCi55 iPSCs (control) and iPSCs carrying the KLF1-ERT2 transgene (iKLF1.2) in the absence (0 nm) and presence (200 nM) tamoxifen. Filtered media is used as a negative control, baseline level. **(C)** Size distribution of particles present in unfiltered conditioned media derived from control (Parental) macrophages and iKLF1.2 macrophages (KLF1) differentiated in the presence (200 nM) and absence (0 nM) of tamoxifen. Graph shows average (and standard deviation) of the different sizes of particles in the respective samples. **(D)** Average size of particles present in conditioned media derived from SFCi55 iPSCs (control) and iPSCs carrying the KLF1-ER^T2^ transgene (iKLF1.2) in the absence (0 nm) and presence (200 nM) tamoxifen with filtered media as control (grey bar). N = 7, statistical test: one-way ANOVA, with Tukey multiple comparison. *p < 0.05, **p < 0.01. The schematic diagram in A was created using Biorender.

We first assessed whether cultured hiPSC-derived macrophages were able to produce EVs and whether their production was altered in response to the activation of KLF1. To ensure the exclusion of any media-derived particles, maturing macrophages from control and iKLF1.2 hiPSCs were switched to pre-filtered media at day 7 of their maturation. Three days later (day 10 of macrophage differentiation), conditioned media was collected and the number and size of EVs were quantified using Nanoparticle Tracking Analysis (NanoSight, Malvern) (Figure 5A). Filtered macrophage-conditioned media was used as a control and as expected, there were virtually no particles detected following filtration (Figure 5B). The concentration of particles in the unfiltered macrophage-conditioned media was highly variable, even between replicate samples (Figure 5B). Although media from macrophages derived from both KLF1.2 and its parental hiPSCs showed a slightly higher concentration of EVs in the presence of tamoxifen, indicating a minor effect of tamoxifen, this trend was not statistically significant (Figure 5B). There was no discernible effect of KLF1 activation on the production of EVs (Figure 5B).

To further characterise the EVs released by hiPSC-derived macrophages, the size distribution of the particles was determined in the conditioned-media derived from each cell line in the presence and absence of tamoxifen (Figure 5C). There was significant variability in the size of detected particles but the most common size of EVs detected was between 150 and 300 nm (Figure 5C). There were no differences in the size distribution of EVs between conditioned media derived from control and KLF1.2 macrophages and the addition of tamoxifen did not have a significant effect (Figure 5D). Taken together these data indicate that the activation of KLF1 had no discernible effect on the number nor the size distribution of EVs produced from hiPSC-derived macrophages.

### The supportive effect of macrophages is reduced when EVs are depleted

Although activation of KLF1 did not alter the number and size distribution of EVs produced from hiPSC-derived macrophages we next assessed whether the EVs generated by KLF1-activated macrophages had any functional difference with respect to their ability to support erythroid cell production and maturation compared to those produced from control macrophages. We considered that the content of EVs might be altered by KLF1 activation and that this might account for some of the enhanced support by KLF1-activated macrophages that we previous reported (Lopez-Yrigoyen et al., 2019).

**FIGURE 6:**
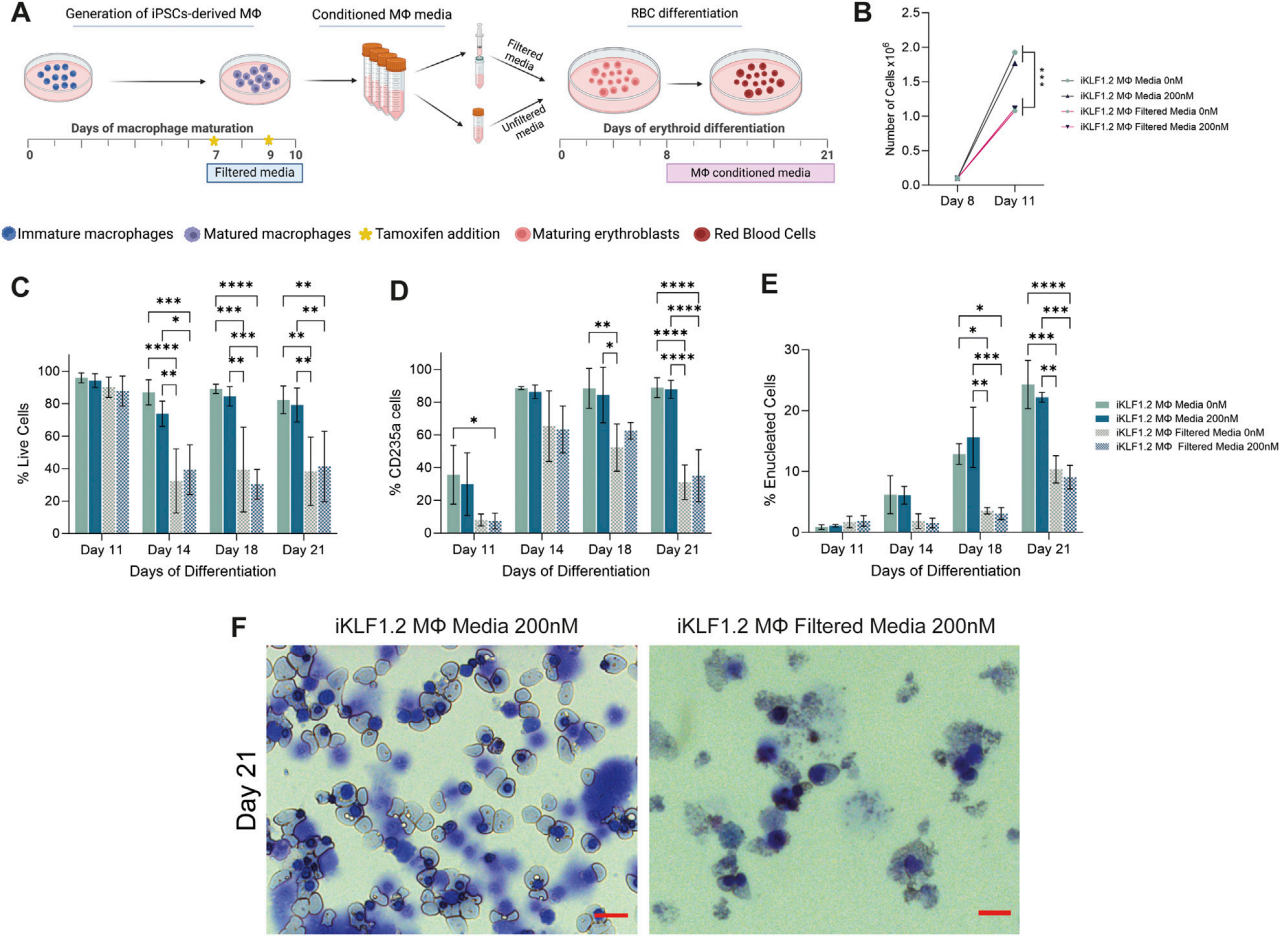
Filtration macrophage conditioned media has a detrimental effect on its supportive role in on red blood cell maturation. **(A)** Schematic diagram of the collection and filtration of conditioned media from iKLF1.2-derived macrophages differentiated without (0 nM) or in the presence of tamoxifen (200 nM) and the subsequent testing of its ability to promote the proliferation and differentiation of umbilical cord blood (UCB) derived erythroid progenitor cells. **(B)** Number of cells (x10^8^) present follow culture for 3 days (day 8–11) in the presence of unfiltered or filtered conditioned media. C-E. Percentage of live cells **(C)**, CD235a+ **(D)** of CD235a^+^CD71-/Hoechst-cells **(E)** cells following culture with unfiltered or filtered conditioned media. **(F)** Representative example of cytospin preparation and H&E staining of cells cultured in unfiltered and filtered conditioned media. N = 3 independent experiments, statistical test: 2-way ANOVA, with Tukey multiple comparison. *p < 0.05, **p < 0.01, ***p < 0.005, ****p < 0.001. The schematic diagram in A was created using Biorender.

Umbilical cord blood derived CD34^+^ haematopoietic progenitor cells were differentiated in the presence of conditioned-media from hiPSC-derived macrophages ± tamoxifen and cell number, viability and differentiation status were assessed by flow cytometry (Figure 6A) (Supplementary Figure S3A). The number and phenotype of erythroid cells that were generated in the presence of EV-depleted conditioned media was compared to unfiltered conditioned media where macrophage-derived EVs were present. The proliferation of cells between days 8 and 11 of the differentiation protocol was lower when cells were cultured in filtered compared to unfiltered media conditioned by iKLF1.2-derived macrophages although activation of KLF1 by tamoxifen had no discernible effect (Figure 6B). The viability of cells at day 11, following culture in unfiltered and filtered macrophage-conditioned media, was comparable indicating that the reduction in cell number at this stage was the result of a reduced rate of proliferation rather than an increase in cell death (Figure 6C). However, as differentiation progressed, the viability of cells was reduced significantly to around 30% when the conditioned media was depleted of EVs by filtration, as compared to unfiltered media. Interestingly this difference was apparent and comparable in erythroid cells that were cultured in conditioned-media from macrophages that were generated from iKLF1.2 hiPSCs irrespectively of tamoxifen addition (Figure 6C) and comparable data were generated when cells were cultured in the presence of the conditioned media from macrophages derived from control hiPSCs (Supplementary Figures S3B, S3C).

As differentiation progressed, there was a significant increase in the proportion of cells expressing the erythroid marker CD235a (Glycophorin A) in all culture conditions (Figure 6D). However, the proportion of cells expressing CD235a was significantly lower when cells were cultured in filtered medium, compared to unfiltered conditioned-media derived from macrophages derived from both iKLF1.2 hiPSC (Figure 6D) and control hiPSCs macrophages (Supplementary Figures S3B–S3D). This suggests that the filtration process excluded components that were associated with the commitment and/or the survival of erythroid cells.

The progression in the differentiation of erythroid cells marked by the expression of CD235a is characterised by the reduction in the expression of CD71 and nuclear extrusion that is marked by the lack of DNA staining (Supplementary Figure S3A). At day 11 of differentiation, all CD235a-expressing cells also expressed CD71 and were nucleated and can therefore be defined as immature erythroid cells (Figure 5E). As differentiation progressed the proportion of nucleated CD235a+ cells that co-expressed CD71 increased significantly, reflecting the progressive production of enucleated erythroid cells. By day 21 of the differentiation process, around 25% of the cells cultured in unfiltered conditioned media were negative for both CD71 and Hoechst, whereas when conditioned media was filtered this proportion was less than 10% (Figure 6E). Morphological analyses of cells at day 21 showed the presence of robust nucleated and enucleated erythroid cells when grown in the presence of unfiltered media compared to the poor quality and fragile cells present following culture in the filtered media (Figure 6F). Comparable data were generated from control hiPSCs (Supplementary Figures S3B–S3D).

It was interesting to note that activation of KLF1 using tamoxifen did not alter the supportive effects of conditioned media in any of the parameters tested (Figures 6B–F), which appears to be in contrast with our findings in co-culture and trans-well assays (Lopez-Yrigoyen et al., 2019). However, we predict that this likely reflects either the stability or the concentration of secreted factors within collected and stored conditioned media compared to the media content in the presence of live macrophages. This question could be addressed in the future by the addition of exogenous growth factors such as ANGPTL7, IL33 and SERPINB2 to the conditioned media.

Taken together, these data indicate that components that do not pass through the 100 nM filter presumably including EVs, support the production, maturation and/or survival of differentiating erythroid cells.

## Discussion

Macrophages associated with the erythroblast island niche *in vivo* play an important role in the production and maturation of red blood cells. However, due to the inaccessibility of this niche there is limited information on its associated cellular and molecular mechanisms processes. Various *in vitro* strategies have proven to be really useful tools but each have their limitations. Although the iPSC strategy used here can generate a limitless supply of genetically engineered macrophages, the data generated from these must be interpreted with caution because it is not clear whether they truly recapitulate EBI behaviour *in vivo*.

We previously demonstrated that activation of the transcription factor KLF1 enhanced the ability of hiPSC-derived macrophages to support erythroid cell proliferation and maturation and we showed that a proportion of that activity was retained within the media that could cross the membrane in a trans-well assay (Lopez-Yrigoyen et al., 2019). That study focussed on the identification of three secreted factors (ANGPTL7, IL33 and SERPINB2) factors that were encoded by KLF1-regulated transcripts and their subsequent validation by the addition of commercially available recombinant factors to differentiating erythroid cells. To uncover mechanisms associated with alternative forms of intercellular interactions within the EBI niche, we set out in this study to identify the translated proteins that were expressed within EBI macrophages and those that were expressed at a higher level within KLF1-activated macrophages.

Proteomic analyses of proteins that were upregulated when KLF1 was activated identified a range of membrane-associated factors that would be predicted to be involved in direct cell-cell interactions between EI-macrophages and developing erythroid cells.

Proteomic analyses of erythroid cells carrying the E325K mutation associated with Congenital Dyserythropoietic Anemia Type IV revealed that the expression level of proteins associated with vesicle tethering were altered compared to control cells (Ferrer-Vicens et al., 2023). In this study we also identified proteins associated with vesicles. For example, we noted that one of the most upregulated proteins was RAB27B which has been reported to play an important role in vesicular trafficking and secretion (Ostrowski et al., 2010). We then demonstrated that filtration of macrophage conditioned media that we presumed would exclude at least a subset of EVs resulted in a reduction in the supportive effects of hiPSC-derived macrophage conditioned-media, providing some functional validation of our proteomic analysis. However, we acknowledge that the filtration process might not remove all EVs and that the process could also result in the exclusion of other factors so further experiments using purified EVs would be required to prove our conclusion. Although there was no discernable effect on the overall production and size distribution of EVs, following KLF1 activation as detected by NTA, our study was limited in that it did not assess the precise phenotype of EVs in detail nor their content. We demonstrated that filtration of conditioned media from KLF1-activated macrophages had a detrimental effect on its supportive effect and that this effect was comparable to filtration from control macrophages. This result, together with the fact that conditioned media from KLF1-activated macrophages has a comparable supportive effect as the control media implies that activation of KLF1 does not alter the functionality of EVs in our system. However, to address directly whether KLF1 activation has a phenotypic effect on the EV phenotype and function would require the isolation and characterisation of EVs from both sources. It would be particularly informative to assess whether isolated EVs support the production and differentiation of erythroid cells and to analyse the proteomic profile of isolated EVs from control and KLF1-activated macrophages (Cianciaruso et al., 2019; Wang et al., 2020).

Given that we had previous shown that KLF1 activation enhanced the supportive role of macrophages, and that part of that activity was independent of cell contact, it was initially surprising to note that activation of KLF1 did not alter the activity of the complete conditioned-media nor the EV-depleted media. Although this appears to contradict what we had previously published, it should be noted that within our previous trans-well assay, any enhancing factors would have been continuously produced by macrophages and that the concentration and/or stability of these factors might not have been retained in conditioned media that is collected and temporarily stored prior to culture with differentiating erythroid cells. The activity of unstable factors or those with a limited half-life would not be retained in conditioned media.

Our previous study showed that some enhancing effect was retained when proliferating and differentiating erythroid cells were separated from supportive macrophages using a 400 nm pore trans-well. We show here that the majority of EVs that are generated by hiPSC-derived macrophages are between 150 and 300 nm so we predict that these would have crossed the filter in our trans-well assay and could have contributed to the enhancing effect. We used 100 nM filters to exclude EVs from conditioned media and we demonstrated that this exclusion resulted in a significant reduction in the production of differentiated erythroid cells. At the early stage of the culture system, there was a significant detrimental effect on the proliferation of erythroid progenitors but, at later stages, this effect was most likely associated with a decrease in the viability of differentiated erythroid cells.

We noted that proteins that were expressed at a higher level when KLF1 was activated were associated with the citric acid cycle, pyruvate metabolism and formation of ATP. It is known that there are significant differences in cellular metabolism associated with different polarised states where classically activated macrophages (also known as M1) rely mostly on glycolysis whereas M2 relies more on oxidative phosphorylation (Viola et al., 2019; Liu et al., 2021). Also, a recent proteomic analyses of different macrophages states identified an enrichment of proteins associated with the TCA cycle and pentose phosphate pathway in M2 polarised macrophages (Oates et al., 2023). Thus, our findings indicate that the metabolic state of KLF1-actvated macrophages was more akin to an M2-like phenotype, consistent with our previous finding that activation of KLF1 in hiPSC-derived macrophages resulted in an increase in phagocytic activity (Lopez-Yrigoyen et al., 2019). It was recently shown that proteins encoded by gene networks associated with mitochondrial biogenesis were upregulated in erythroid cells carrying the E325K mutation that is associated with Congenital Dyserythropoietic Anemia Type IV (Ferrer-Vicens et al., 2023). Proteomic analysis of macrophages carrying this pathogenic mutation will reveal whether comparable changes are associated with the EI niche using our recently developed model of this disease (May et al., 2023).

## Data Availability

The original contributions presented in the study are included in the article/Supplementary Materials. The mass spectrometry proteomics data have been deposited to the ProteomeXchange Consortium via the PRIDE (Perez-Riverol et al., 2022) partner repository with the dataset identifier PXD050357. Further inquiries can be directed to the corresponding author.
