# Supplementary material for: Proteomic analysis reveals a potential role for extracellular vesicles within the erythroblastic island niche
Source: Front Mol Biosci. 2024 Apr 16;11:1370933. doi: 10.3389/fmolb.2024.1370933 (PMC11058792; doi:10.3389/fmolb.2024.1370933)
Supplement: Supplementary file 1 [file Table1.docx]

**Supplementary Tables**

**Supplementary Table 1.** List of conjugated antibodies used for flow cytometry analysis and respective clones, company, catalogue number and used dilution.

| **Marker** | **Fluorochrome** | **Clone** | **Company** | **Cat. number** | **Dilution** |
| --- | --- | --- | --- | --- | --- |
| CD45 | FITC | HI30 | eBioscience | 11-0459-42 | 1:100 |
| CD93 | PE | VIMD | Biolegend | 336107 | 1:200 |
| 25F9 | eF660 (APC) | eBio25F9 | eBioscience | 50-0115-42 | 1:25 |
| CD169 | APC | 7-239 | Biolegend | 346008 | 1:40 |
| CD163 | PE-Cy7 | GHI/61 | BioLegend | 333614 | 1:40 |
| CD235a | FITC | HIR2 | eBioscience | 11-9987-80 | 1:200 |
| CD71 | APC | OKT9 | eBioscience | 17-0719-42 | 1:200 |
| NucBlue | Hoechst |  | Thermo Fisher | R37605 | 1:50 |
| Live/Dead | IR |  | Thermo Fisher | L10119 | 1:100 |

**Supplementary Table2.** Primers used for qPCR analysis with respective Forward and Reverse sequences and efficiency.

| **Primers** | **Sequence - Forward** | **Sequence - Reverse** | **Primer**  **Efficiency** |
| --- | --- | --- | --- |
| FDCSP | GTCAGAGAGAAAGAACTGACTGAAAC | GAGACTGGGAAACCAACAGC | 1.950 |
| PI16 | TCCTTCCATTCCTGAGTCCA | AGTCCTTCCCACACAAGCAC | 2.000 |
| IL33 | GTGCTTAGCATGTGTGGAATG | GATGCAGTTATACAGAGGGAAATAAA | 1.890 |
| CD11B | GGCATCCGCAAAGTGGTA | GGATCTTAAAGGCATTCTTTCG | 1.880 |
| PLAUR | GTAGCCACCGGCACTCAC | TGGTCTCAGGGCAGTAGTACTTT | 1.963 |
| GAPDH | AGCCACATCGCTCAGACAC | GCCCAATACGACCAAATCC | 2.010 |
| β-ACTIN | CCAACCGCGAGAAGATGA | CCAGAGGCGTACAGGGATAG | 1.980 |
