# Supplementary material for: Proteomic analysis reveals a potential role for extracellular vesicles within the erythroblastic island niche
Source: Front Mol Biosci. 2024 Apr 16;11:1370933. doi: 10.3389/fmolb.2024.1370933 (PMC11058792; doi:10.3389/fmolb.2024.1370933)
Supplement: Supplementary file 2 [file Image1.pdf]

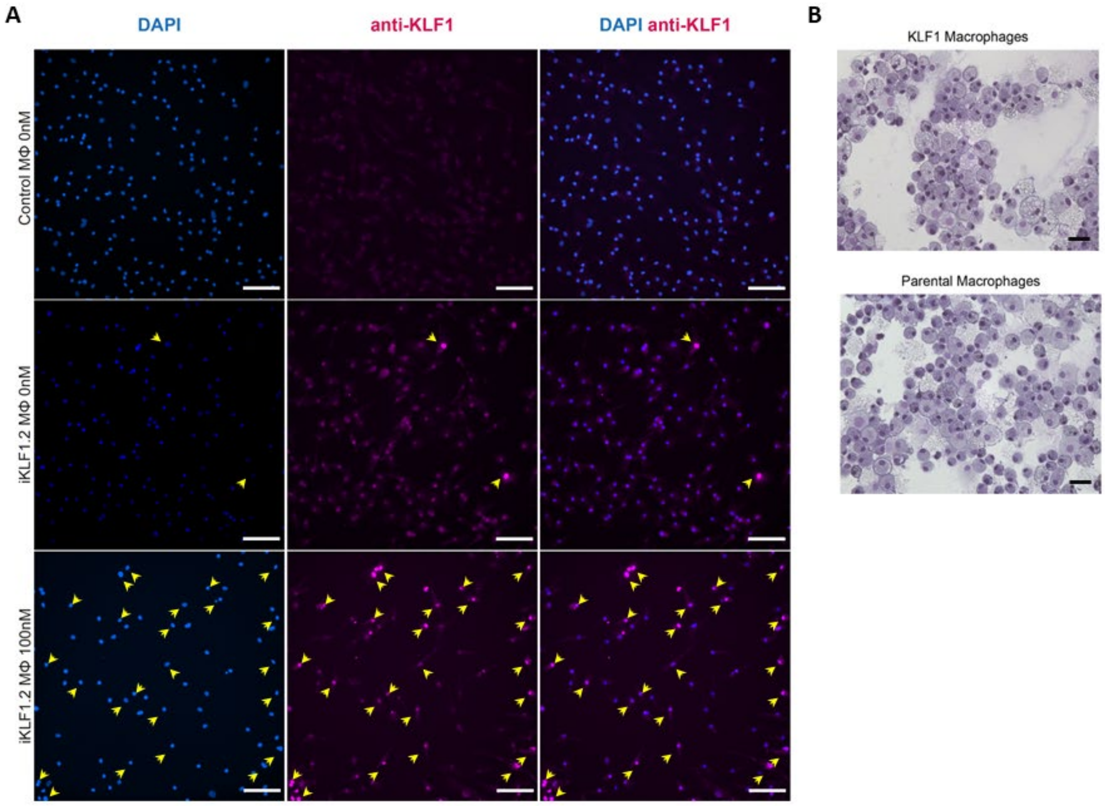


**Supplementary Figure S1.**

A. Immunohistochemistry of macrophages derived from SFCi55 hiPSCs (control/ parental macrophages) and from iKLF1.2 hiPSCs without tamoxifen (0nM) and with 100nM of tamoxifen (100nM). Cells were stained with DAPI (blue) to identify nuclei and with an anti-KLF1 antibody (magenta). Single and merged images are shown. Yellow arrows indicate KLF1-stained nuclei. (scale bar 100µm).
B. Macrophages derived from control/parental and iKLF1.2 macrophages show comparable morphology (scale bar 100µm).

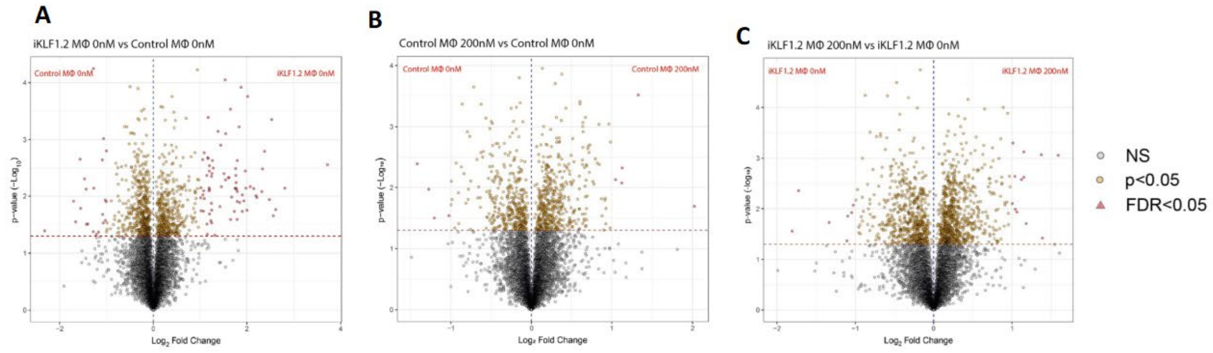


**Supplementary Figure S2.**
Volcano plots of sample comparison between iKLF1.2 0nM vs control 0nM (A), Control 200nM vs Control 0nM (B), iKLF1.2 200nM vs iKLF1.2 0nM (C).

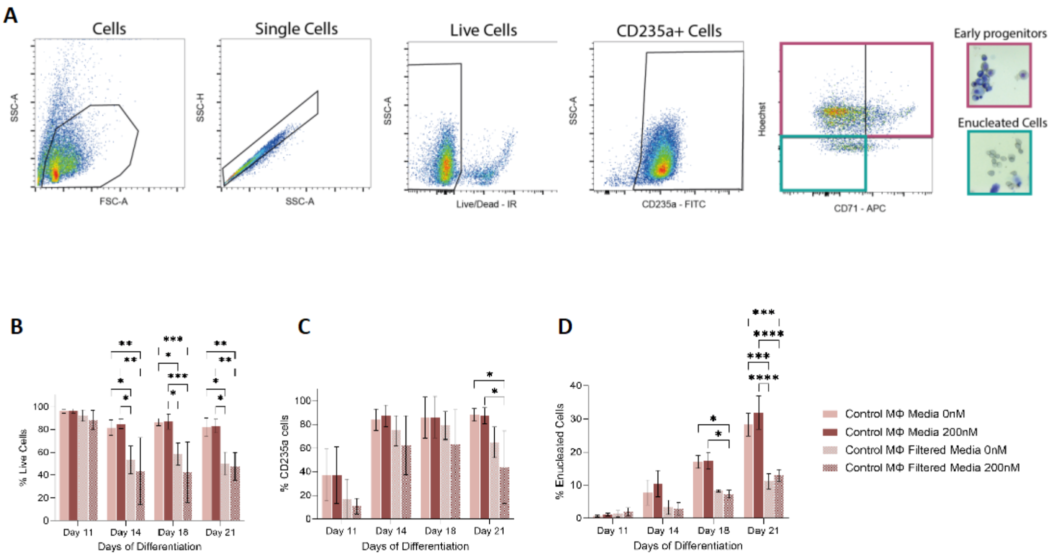


**Supplementary Figure S3.**

A. Flow cytometry strategy for the analyses of differentiating erythroid cells. Cells of interest were gated from the whole population acquired, followed by single, live and CD235a+ cell selection. Maturation and enucleation were assessed by the lack of expression of both CD71 and Hoechst DNA staining cells in contrast with progenitor cells that are still nucleated, and therefore positive for Hoechst. B-D. Percentage live (B), CD235a$^{+}$ (C) and CD235a$^{+}$, CD71$^{-}$, Hoescht$^{-}$ cells generated from UCB progenitors at 11, 14, 18 and 31 days of maturation in the presence of unfiltered or filtered media derived from control iPSC-derived macrophages in the absence (0nM) or presence of 200nm tamoxifen.

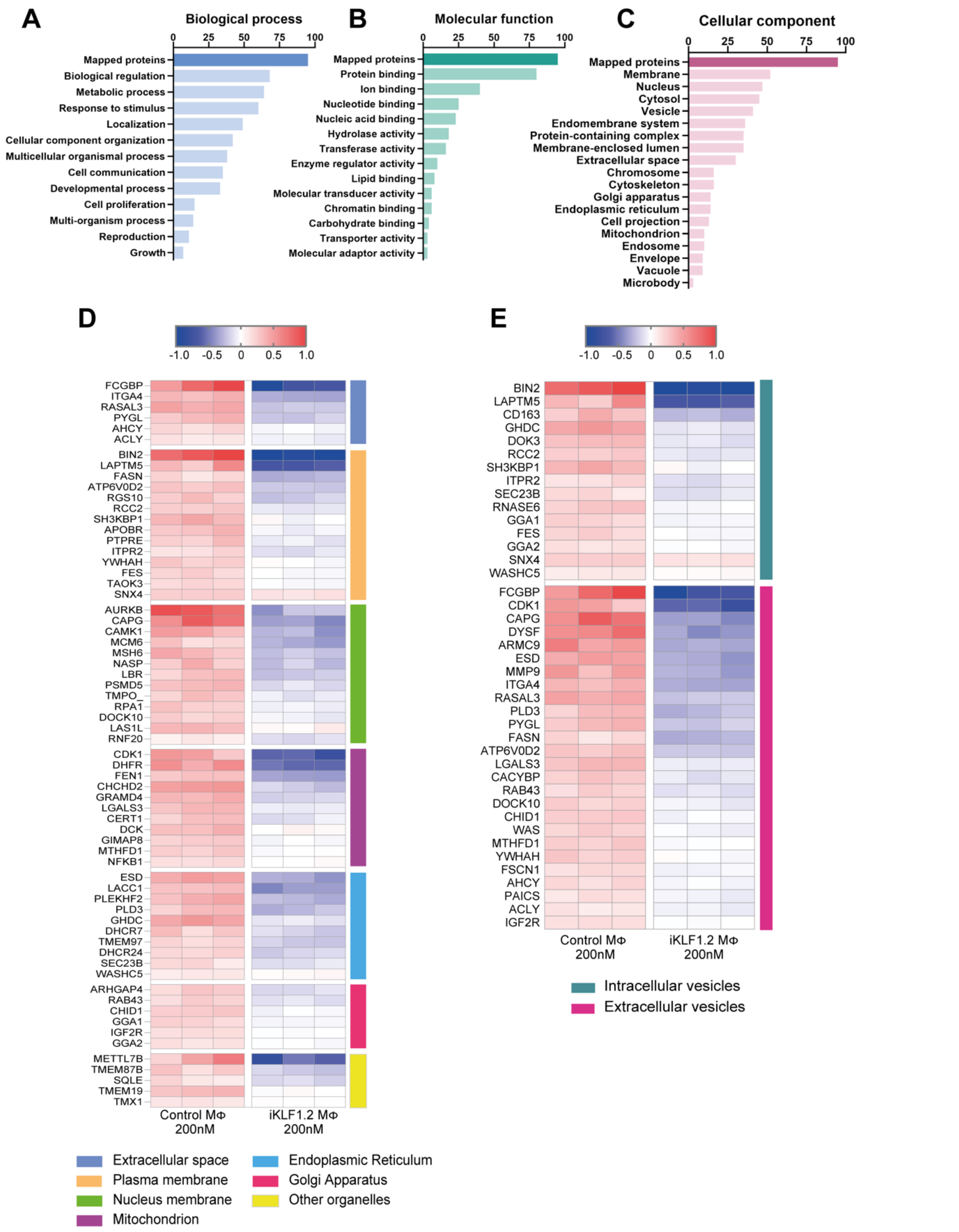


Supplementary Figure S4.
A-C. Gene Ontogeny (GO) analysis of downregulated proteins in KLF1 activated macrophages showing the biological processes (A), molecular function (B) and cellular component of the protein subset (C) . GO analysis was carried using Web Gestalt analysis tool.
D-E Heatmap representation of significantly downregulated proteins in KLF1 activated macrophages associated with membrane complexes of the cells, divided in proteins from extracellular space, plasma membrane, nucleus membrane, mitochondrion, endoplasmic reticulum, Golgi apparatus and

other smaller organelles (D) . Proteins associated with  vesicles where also identified and divided in proteins from intracellular and extracellular vesicles (E).

Student t-test was used as statistical analysis of the differentially expressed proteins between samples, with p-value >0.05.Z-score between -1 and 1 was calculated to represent the differences between macrophages. Proteins shown in the heatmap represent proteins with  -0.4≤Fold Change≥0.4.

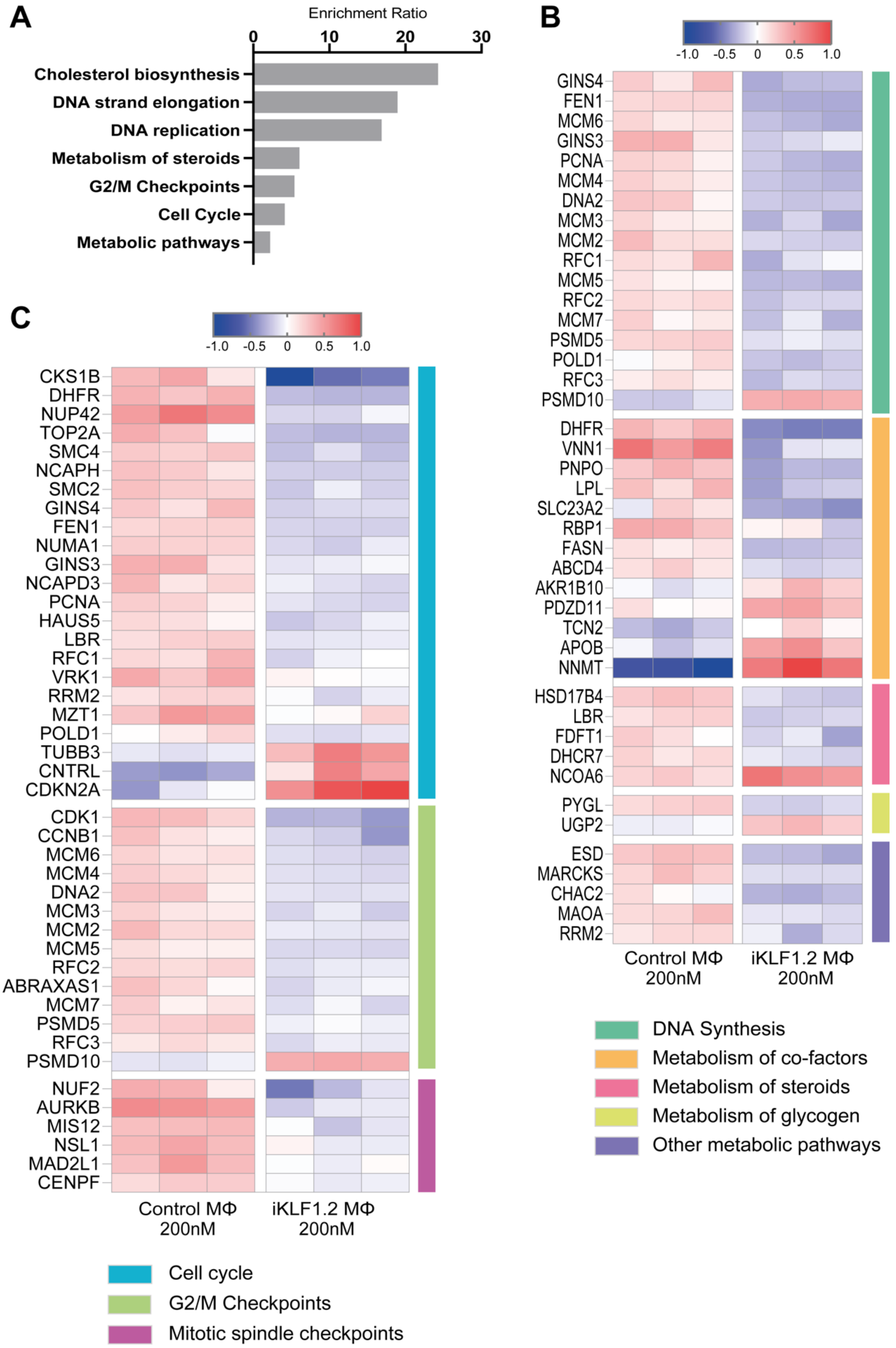


Supplementary Figure S5

A <u>Cellular pathways downregulated upon activation of KLF1 in macrophages.</u> Graph represents the enrichment ratio of the downregulated proteins in specific biological pathways. Cellular pathways

downregulated in KLF1 activated macrophages with high enrichment analysis, where metabolic pathways are the most represented. Analysis was carried using Reactome tool.

B-C. Heatmap representation of significantly expressed proteins in KLF1 activated macrophages associated with metabolic pathways such as DNA synthesis, metabolism co-factors, metabolism of steroids, metabolism of glycogen and other downregulated metabolic pathways (B). Downregulated proteins associated with cell cycle, G2/M checkpoint and mitotic spindle (C).

Student t-test was used as statistical analysis of the differentially expressed proteins between samples, with p-value >0.05. Z-score between -1 and 1 was calculated to represent the differences between macrophages. Proteins shown in the heatmap represent proteins with -0.4≤Fold Change≥0.4.
